# Translation of the UK Pediatric Influenza Vaccination Programme in Primary Schools to 13 European Countries Using a Dynamic Transmission Model

**DOI:** 10.36469/9802

**Published:** 2017-08-18

**Authors:** Laetitia Gerlier, Judith Hackett, Richard Lawson, Sofia Dos Santos Mendes, Martin Eichner

**Affiliations:** 1 QuintilesIMS, Real-World Evidence Solutions, Zaventem, Belgium; 2 AstraZeneca, Gaithersburg, MD, USA; 3 AstraZeneca, Brussels, Belgium (Current affiliation: MSD, Brussels, Belgium); 4 Institute for Clinical Epidemiology and Applied Biometry University of Tübingen, Tübingen, Germany; Epimos GmbH, Dusslingen, Germany

**Keywords:** europe, dynamic transmission model, indirect protection, live-attenuated influenza vaccine, pediatric vaccination, seasonal influenza

## Abstract

**Objectives:** To simulate the impact of a pediatric influenza vaccination programme using quadrivalent live attenuated influenza vaccine (QLAIV) in Europe by applying coverage rates achieved in the United Kingdom during the 2014–2015 season and to compare the model outcomes to the UK results.

**Methods:** We used a deterministic, age-structured, dynamic transmission model adapted to the demography, contact patterns and influenza incidence of 13 European countries, with a 10-year horizon. The reference strategy was the unchanged country-specific coverage rate, using quadrivalent inactivated vaccine (assumed efficacy against infection from 45% in 1-year-old children to 60% in healthy adults). In the evaluated strategy, 56.8% of 5–10-year-old children were additionally vaccinated with QLAIV (assumed efficacy 80%), as was the case in 2014–2015 in the United Kingdom’s primary school pilot areas. Symptomatic influenza cases and associated medical resources (primary care consultations [PCC], hospitalization, intensive care unit [ICU] admissions) were calculated. The evaluated versus reference strategies were compared using odds ratios (ORs) for PCC in the target (aged 5–10-years) and non-target adult (aged >17 years) populations as well as number needed to vaccinate (NNV) with QLAIV to avert one PCC, hospitalization or ICU admission. Model outcomes, averaged over 10 seasons, were compared with published real-life data from the United Kingdom for the 2014–2015 season.

**Results:** Over 13 countries and 10 years, the evaluated strategy prevented 32.8 million of symptomatic influenza cases (172.3 vs 205.2 million). The resulting range of ORs for PCC was 0.18–0.48 among children aged 5–10-years, and the published OR in the United Kingdom was 0.06 (95% confidence interval [0.01; 0.62]). In adults, the range of ORs for PCC was 0.60–0.91 (UK OR=0.41 [0.19; 0.86]). NNV ranges were 6–19 per averted PCC (UK NNV=16), 530–1524 per averted hospitalization (UK NNV=317) and 5298–15 241 per averted ICU admission (UK NNV=2205).

**Conclusions:** Across a range of European countries, our model shows the beneficial direct and indirect impact of a paediatric vaccination programme using QLAIV in primary school-aged children, consistent with what was observed during a single season in the United Kingdom. Recommendations for the implementation of pediatric vaccination programmes are, therefore, supported in Europe.

## INTRODUCTION

Among countries that have implemented an influenza vaccination programme including children (Canada, Finland, United Kingdom, United States), two main indicators are used to assess the performance of the newly implemented vaccination campaign: the coverage rate reached in the target population and the number of prevented clinical events in the total population. The former outcome can be estimated via surveys or administrative methods,[1] while the latter implies a comparison of the number of clinical events before and after changing the coverage rate. Canadian and US studies analyzing a sufficiently long period of time demonstrated substantial reduction of the influenza-related morbidity through a universal influenza vaccination programme including healthy children.[2,3] Other studies focusing on a single influenza season demonstrated both direct benefits of pediatric vaccination in the target-population and indirect benefits in the general population.[4-8] A systematic literature review confirmed that the vaccination of healthy children against influenza provides both health benefits to the children themselves and economic benefits to the community.[9] The authors however highlight the difficulty in measuring these effects, and the need for further research. As a complement to observational studies, modelling exercises were also useful to compare different vaccination stategies. Dynamic transmission models, considering direct and indirect protection in a population, generally emphasized large protection effects,[10-13] yet the question must be addressed whether the magnitude of protective effects is reflective of real life as transmission models must invariably be based on simplifying assumptions. In our study, we used a dynamic transmission model available for 13 European countries[1,14-18] to simulate the UK 2014–2015 pediatric vaccination programme and compare model outcomes to observed, real-life results.

## AIMS AND OBJECTIVES

We simulate the impact of a pediatric influenza vaccination programme using a quadrivalent live attenuated influenza vaccine (QLAIV) by applying coverage rates and outcomes achieved in the United Kingdom during the 2014–2015 season to 13 European countries, including Austria, Belgium, Finland, France, Germany, Greece, Italy, Luxembourg, The Netherlands, Poland, Portugal, Spain, and Sweden. A secondary objective was to compare the direct and indirect impact of the vaccination programme from the model with the UK results.

## METHODS

### Study Design

A deterministic, age-structured, dynamic transmission model was used to simulate the transmission of influenza in the population and to compare different vaccination strategies including direct and indirect protection effects. The model simulates the independent transmission of four influenza viruses strains: A(H1N1), A(H3N2), one B strain coming from the B/Victoria lineage, and one B strain coming from the B/Yamagata lineage. Demographic changes and transmission dynamics are described by a system of interacting differential equations. Contact patterns between individuals (ie, average age-dependent numbers of contacts per person per day) were derived from the European Polymod study, using the matrix for physical and non-physical contacts.[19] The all-year average of the basic reproduction number $R_0$, representing the number of secondary infections produced by a single infected case in a fully susceptible population, was calibrated to country-specific reported incidence data for laboratory-confirmed influenza, averaged over two or more seasons. $R_0$ was assumed to vary throughout the year: it was 43% higher than average around Christmas and 43% lower in summer.[11] Model inputs and assumptions are presented in Table 1 (values common to all countries) and Table 2 (country-specific values). Further details on the model design and methods were described in a previously published study.[16]

**Table 1.** Simulation Tool Input Values, Common to All Countries

| Parameter | Age Group (years) | Value Base Case | | Source |
|---|---|---|---|---|
| **Population, total 13 countries (average 2015–2025)** | **All ages** | **368.76 million individuals** | | **EuroStat** |
| **Population with risk factors** | | | | |
| % high-risk, without severe asthma | 0–17 | 14.3% of age group | | 20, 58 |
| | 18–44 | 16.1% of age group | | 20, 58 |
| | 45–64 | 32.1% of age group | | 20, 58 |
| | >65 | 100% of age group | | 20, 58 |
| % high-risk, with severe asthma | 0–17 | 1.8% of age group | | 21 |
| **Natural history** | | | | |
| External infection rate | All ages | 1/1,000 per person per year, fluctuating seasonally, peak in February | | Assumption |
| Proportion of infected developing symptoms | All ages | 66.9% | | 59 |
| Mean duration of latency | All ages | 1 day | | 59 |
| Mean duration of contagiousness | All ages | 5 days | | 59 |
| Mean duration of natural immunity after infection | All ages | Influenza A: 6 years<br>Influenza B: 12 years | | 11 |
| **Vaccine efficacy estimates (all strains)** | | QIV | QLAIV | |
| | 1 y | 45% | NA | 50 |
| | 2–17 y | 59% | 80% | 50 |
| | Adults >18, low-risk | 60% | NA | 51 |
| | Adults >18, high-risk | 58% | NA | 52 |
| **Immunity duration after vaccination** | | QIV | QLAIV | |
| % immunity loss at end of 1st season | All ages | 100% | 30% (2nd season: 100%) | 48 |
| **Re-vaccination preference factor** | All ages | RR=6.0 of being vaccinated, when vaccinated in previous year[a] | | 39 |
| **Vaccination strategies** | | | | |
| Reference strategy | | Current coverage rate with QIV, country-specific | | See Table 2 |
| Evaluated strategy | <4 low-risk | unchanged | 0% | |
| | 5–10 low-risk | 0% | 56.8%[b] | 8 |
| | 11–17 low-risk | unchanged | 0% | |
| | >18 low-risk | unchanged | 0% | |
| | 5–10 high-risk, no severe asthma | unchanged | 56.8%[b] | 8 |
| | 11–17 high-risk | unchanged | 0% | |
| | >18 high-risk | unchanged | 0% | |
| | ≥ 65 | unchanged | 0% | |
| | 5–10 high-risk with severe asthma | 56.8%[b] | 0% | 8 |

**Table 1.** Simulation Tool Input Values, Common to All Countries (continued)

| Parameter | Age Group (years) | Value Base Case | | Source |
|---|---|---|---|---|
| | | Low risk | High risk | |
| **Medical resources used in case of symptomatic influenza** | | | | |
| | 1 | 67% | 67% | 49, 50 |
| | 2–64 | 33% | 67% | 49, 50 |
| | ≥ 65 | NA | 67% | 49, 50 |
| | 1 | 1.0% | 1.0% | 49, 52 |
| | 2–64 | 0.2% | 1.0% | 49, 52 |
| | ≥ 65 | NA | 3.0% | 49, 52 |
| | 1 | 0.1% | 0.1% | 53 |
| | 2–64 | 0.02% | 0.1% | 53 |
| | ≥ 65 | NA | 0.3% | 53 |

ICU: Intensive care unit; NA: not applicable; PCC: Primary care consultation; QLAIV: quadrivalent live-attenuated influenza vaccine; QIV: quadrivalent inactivated vaccine; RR: relative risk

[a]Odds ratio (OR) 30–60 converted into relative risk (RR) using the original research[39]

[b]Maximum coverage reached in three annual steps

Legend: reminder of main model characteristics:

- Population subdivided into 1-year age cohorts and risk classes
- "High-risk" group: all individuals aged ≥ 65 years and individuals from 6 months of age with immunodeficiency or any chronic cardiovascular, hepatic, renal, metabolic, neurological, or pulmonary comorbidity[58]
- Contact patterns between individuals (i.e. average age-dependent numbers of contacts per person per day): derived from the European Polymod study, using the matrix for physical and non-physical contacts.[19] Contact data from a neighbouring country was used in absence of country-specific information.
- Independent transmission of four influenza viruses strains: A(H1N1), A(H3N2), one B strain coming from the B/Victoria lineage, and one B strain coming from the B/Yamagata lineage
- The all-year average of the seasonally fluctuating basic reproduction number $R_0$ calibrated to country-specific reported incidence data for laboratory-confirmed influenza, averaged over two or more seasons:

Austria, Finland, Germany, Sweden: influenza infection incidence[60,61]

Greece, Italy, The Netherlands, Poland: influenza symptomatic cases[62-64]

Belgium, France, Luxembourg, Portugal, Spain: influenza physician visits[51,65-67]
- The basic reproduction number $R_0$, representing the number of secondary infections produced by a single infected case, was assumed to vary over the year: it was 43% higher than average around Christmas and 43% lower in summer.[11]
- The same value of the $R_0$ was used for each one of the four influenza strains which were assumed to be transmitted independently.
- Model calibrations reproduced country-specific incidence targets with error rates below 1%, leading to values of the all-year average of $R_0$ which ranged from 0.90 (implying an $R_0$ peak value around Christmas of 1.29) to 1.28 (peak value 1.83) across countries.
- During the simulations, the entire population was assumed to be further exposed to an external infection rate of 1 per 1000 susceptible population-years, which also fluctuated seasonally.
- Immunity patterns of the simulated population were initialized during 20 years using the observed vaccination coverage
- Vaccinations were assumed to be performed annually from October 1 to November 30.

**Table 2.** Simulation Tool Input Values, Country-specific

| Parameter | AU | BE | FI | FR | GE | GR | IT | LU | NL | PL | PT | SP | SW |
|---|---|---|---|---|---|---|---|---|---|---|---|---|---|
| Population (million)[1] | 8.77 | 11.80 | 5.60 | 67.45 | 80.50 | 10.71 | 61.87 | 0.63 | 17.10 | 38.28 | 10.19 | 45.75 | 10.11 |
| % aged <18 | 17.5% | 20.7% | 19.9% | 21.8% | 15.7% | 16.9% | 16.7% | 20.8% | 19.8% | 18.1% | 16.4% | 17.2% | 21.0% |
| % growth 2014–25 | 6.3% | 9.8% | 5.6% | 4.8% | –0.4% | –5.8% | 3.7% | 28.8% | 3.2% | –1.1% | –4.5% | –3.1% | 9.7% |
| Contact matrix[2] | GE* | BE | FI | BE* | GE | IT* | IT | LU | NL | PL | IT* | IT* | FI* |
| **Number of contacts[a]** | | | | | | | | | | | | | |
| Children-Children | 1.67 | 2.73 | 3.24 | 2.91 | 1.51 | 5.57 | 5.39 | 5.22 | 5.27 | 4.27 | 1.67 | 2.73 | 3.24 |
| Children-Adults | 0.81 | 1.12 | 0.95 | 1.08 | 0.77 | 2.34 | 2.35 | 2.29 | 1.54 | 1.43 | 0.81 | 1.12 | 0.95 |
| Children-≥ 65 | 1.03 | 1.50 | 1.24 | 1.60 | 0.98 | 1.94 | 1.92 | 1.67 | 1.37 | 1.59 | 1.03 | 1.50 | 1.24 |
| Adults-Adults | 0.64 | 1.18 | 0.60 | 1.14 | 0.64 | 1.82 | 1.83 | 1.38 | 0.95 | 1.13 | 0.64 | 1.18 | 0.60 |
| Adults-≥ 65 | 3.99 | 5.83 | 5.33 | 5.62 | 3.91 | 8.54 | 8.52 | 8.61 | 6.91 | 8.33 | 3.99 | 5.83 | 5.33 |
| ≥ 65-≥ 65 | 1.36 | 1.67 | 1.27 | 1.78 | 1.61 | 3.31 | 3.37 | 1.53 | 1.97 | 2.34 | 1.36 | 1.67 | 1.27 |
| Total per individual | 9.51 | 14.04 | 12.62 | 14.13 | 9.41 | 23.52 | 23.38 | 20.70 | 18.00 | 19.09 | 9.51 | 14.04 | 12.62 |
| **Influenza vaccination coverage (current, QIV)** | | | | | | | | | | | | | |
| 0–17, low-risk | 6.7–9.7 | 3.5 | 5–13 | 1.6 | 4.2 | 1.4 | 2.2–4.5 | 1.5 | 1.5 | 1.4–2.4 | 1.8 | 2.3 | 1-2 |
| 0–17, high-risk | 18.4 | 12.6 | 24 | 17.3–42.9 | 22.6 | 18.4 | 52.4 | 4.2 | 34.8 | 7.4 | 31 | 15.7 | 8.0 |
| 18–64, low-risk | 8.9 | 11.3–18.9 | 6.2 | 6.0–21.0 | 2.0–15.0 | 6.3 | 3.4–12.0 | 4.2–13.3 | 7.3–7.4 | 3.1 | 3.0–4.9 | 2.8 | 1.1–4.2 |
| 18–64, high-risk | 18.4 | 22.3-32.6 | 24.0 | 31.9 | 22.6 | 18.4 | 38.0 | 4.2–13.3 | 40.6–52.5 | 7.4 | 31.0 | 18.7 | 1.1–4.2 |
| ≥ 65 | 38.1 | 58.0 | 34.0 | 54.0 | 50.6 | 18.4 | 62.7 | 52.4 | 74.3 | 7.4 | 45.0 | 57.0 | 45.8 |
| *Sources* | 23,25 | 26 | SW* | 28,68 | 29,30 | 31,69 | 8,24 | BE* | 33 | 34 | 35 | 36 | 37 |
| **Calibration target** | | | | | | | | | | | | | |
| Target endpoint | GE* | Visits | SW* | Visits | Incidence | IT* | Cases | BE* | Cases | Cases | Visits | Visits | Incidence |
| Seasons / years | | 2003–2009 | | 2005–2012 | 2006–2007 | | 2009–2012 | | 2005–2007 | 2010–2011 | 2000–2003 | 2000–2009 | 2012–2013 |
| Timeframe | | Wk 40 to Wk 20 | | Wk 1 to Wk 10 | Wk 40 to Wk 20 | | Wk 40 to Wk 20 | | Full year | Wk 40 to Wk 20 | Wk 40 to Wk 20 | Wk 1 to Wk 10 | Wk 40 to Wk 20 |
| Average value | 10.6% | 285 951 | | 1 272 857 | 10.6% | | 2 510 489 | | 410 200 | 133 167 | 55 687 | 422 978 | 10.0% |
| Min value | 7.5% | 212 195 | | 800 000 | 9.5% | | 2 330 301 | | 338 500 | 0 | 29 876 | 311 668 | 7.5% |
| Max value | 22.4% | 363 880 | | 2 150 000 | 11.7% | | 2 759 959 | | 518 600 | 0 | 71 282 | 592 169 | 22.4% |
| Estimated influenza infection incidence (%) | | 8.9% [6.6–11.5] | | 7.7% [4.9–12.7] | | | 6.3% [5.9–7.0] | | 3.7% [3.1–4.8] | 3.8% | 2.8% [1.5–4.6] | 3.9% [2.9–5.5] | 2012–2013 |
| *Sources* | GE* | 65 | SW* | 51 | 60 | IT* | 62 | BE* | 63 | 64 | 66 | 67 | 61 |
| Basic reproduction number ($R_0$)[b] | 1.12 | 1.18 | 1.10 | 1.28 | 1.12 | 1.05 | 1.05 | 1.10 | 0.90 | 1.05 | 0.92 | 1.02 | 1.11 |

AU: Austria; BE: Belgium; FI: Finland; FR: France; GE: Germany; GR: Greece; IT: Italy; LU: Luxembourg; NL: The Netherlands; PL: Poland; PT: Portugal; SP: Spain; SW: Sweden

1: EuroStat 2015 – population size calculated at the mid-point of years 2014 and 2025 population (N=368.76 million over 13 countries)

2: Mossong et al 2008[19]

*: Assumption, using available information from neighbouring country in absence of local data at the time of our analysis a: Average number of contact per day, taking into account contact matrix and demographic structure of the country b: Calibration outcome, error <1% between average target value and model output value

### Compared Vaccination Strategies

The reference strategy was the unchanged country-specific coverage rate, using quadrivalent inactivated vaccine (QIV). In most European countries, influenza vaccination policies target only individuals with high risk from 6 months of age – with chronic disease/immune deficiency and/or aged individuals of ≥ 65 years – representing approximately 180 million individuals (36%) overall, in the European population.[20] The proportion of high-risk individuals increased with age in the model: 16% of children (of whom 11% are ineligible to receive QLAIV due to a severe form of asthma[21]), 16% of 18–44-year-olds, 32% of 18–64-year-olds and 100% of those aged ≥ 65 years by definition (Table 1). In the evaluated strategy of our simulations, children aged 5–10 years were additionnally vaccinated using QLAIV with a coverate rate of 56.8%, as was the case in 2014–2015 in the United Kingdom's primary school pilot areas. Children suffering from a severe form of asthma continued to receive QIV in our model, with new coverage of 56.8%. Using the model, both strategies were evaluated during 10 influenza seasons, starting in 2015–2016. Current vaccination coverage rates per age-risk group and country were derived from the most recent reports by the European Centre for Disease Prevention and Control (ECDC),[1,22] from multi-country surveys,[23,24] and from country-specific studies[13,25-38] (Table 2). According to a study by the French sick fund,[39] individuals vaccinated in a given year had a higher probability of being re-vaccinated the following year (odds ratio [OR] 30–60). A preferential re-vaccination factor was implemented in the simulations accordingly for all age groups.

### Vaccination Properties

The vaccine efficacy against influenza infection in children aged 2–17 years, assessed in meta-analyses of randomized controlled trials, was 59% (95% confidence interval [CI] [41–71%]) for the trivalent inactivated vaccine and 80% [68–87%] for the trivalent live-attenuated vaccine.[40] The trivalent inactivated vaccine showed an efficacy of 60% [53–66%] in healthy adults,[41] and 58% [34–73%] in the population aged >65 years.[42] The latter efficacy value was applied to all high-risk individuals using the inactivated vaccine. In the model, we assumed the efficacy of quadrivalent vaccines to be the same as that of the trivalent ones reported in the meta-analyses.[43-45] The duration of vaccination-acquired immunity is known to wane quickly after vaccination with an inactivated vaccine;[46,47] consequently, all QIV-acquired immunity was assumed to be lost after one influenza season. Immunity acquired by live-attenuated vaccination may last at least until the following season: according to an Asian study, 70% of the vaccinees who were successfully immunized in the first year with a live-attenuated vaccine were also protected in the second year against matched strains without re-vaccination.[48] Accordingly, we assumed that 30% of the immunity acquired by QLAIV vaccination was lost at the end of the first influenza season, whereas the remaining part was lost after the second season (Table 1).

### Medical Resources

Based on the simulated number of symptomatic influenza cases, the probability of requiring a primary care consultation (PCC), a hospitalization or an admission to the intensive care unit (ICU) was applied to estimate the total number of influenza-related medical resources used, with the reference and the evaluated strategies. Country-specific data regarding the management of influenza symptoms were consulted[49-53] and high-level estimates of the medical resources probabilities were applied to all countries: the rate of PCC was assumed to be 33% in the general population and 67% for 0–1 year-old children and high-risk individuals, the rate of hospital admission was assumed to be 0.2% in the general population, 1.0% for children aged 0–1 years and high-risk individuals aged 2–64 years and 3.0% for those aged ≥ 65 years. Further, ICU admissions were assumed to occur in 10% of hospitalization cases (Table 1).

### Model Outcomes

The number of symptomatic cases cumulated over the 10-year evaluation period was expressed as a number of cases per 100 000 population-years (total number of events divided by the model time horizon ie, 10 years and by the average population size over the 10-year evaluation period, then multiplied by 100 000). The incidence was estimated in the total population and separately in the subgroup of children aged 5–10 years (targeted population) and in adults aged ≥18 years. These calculations were applied to each country separately, and to the 13 countries pooled together.

The evaluated and reference strategies were then compared in terms of medical resources, using the same statistics as those calculated for the UK influenza season 2014–2015: OR for PCC in the target population (children aged 5–10 years) and the non-targeted population of adults aged ≥18 years, and numbers needed to vaccinate (NNV) with QLAIV to avert one PCC, one hospitalization or one ICU admission, respectively. The model outcomes were compared with published real-life data from the United Kingdom.[8]

### Sensitivity Analyses

In a sensitivity analysis, the OR and NNV were estimated after varying the rate of medical resources used in case of symptomatic influenza (PCC, hospitalizations and ICU), using ±25% variations around the base case probabilities.

Sensitivity analyses regarding the vaccine efficacy, basic reproduction number and immunity duration were performed elsewhere, based on the same version of the simulation model.[18]

## RESULTS

In the following section and unless otherwise specified, the central value is obtained after cumulating the cases in 13 countries, and the ranges indicate the minimum and maximum values encountered for the 13 modelled countries.

### Impact on Symptomatic Cases

When considering QLAIV vaccination coverage of 56.8% of children aged 5–10 years, the absolute number of symptomatic influenza cases dropped from 205.2 (reference scenario) to 172.3 million (evaluated scenario) over 10 seasons in 13 countries (N=368.76 million inhabitants on average over 2015–2025). This corresponds to 32.8 million prevented symptomatic influenza cases or 891 prevented cases per 100 000 population-years in 13 European countries. Across countries, the number of symptomatic influenza cases of any age prevented by QLAIV vaccination ranged from 454 to 1663 cases per 100 000 annually (lowest and highest values observed across 13 countries). Absolute numbers of cases per country are shown in Supplementary material S1.

### Odds Ratio Primary Care Consultation

As a consequence of the prevented symptomatic cases, the number of influenza-related PCC cumulated over 10 years in the total population dropped from 86.3 million (2341 per 100 000 population-years) to 72.9 million (1977 per 100 000 population-years) ie, by 13.4 million (364 per 100 000 population-years). In the target population of children aged 5–10 years, the reduction amounted to 115 PCC per 100 000 population-years (Table 3), ranging from 47 to 218 PCC per 100 000 across 13 countries. The corresponding OR for PCC

among 5–10 year-olds was 0.38 (Table 1) and ranged from 0.18 to 0.48 across the simulated countries, while the published OR in the United Kingdom was 0.06 with a 95% CI of [0.01; 0.62] (Figure 1).

In adults aged ≥18 years, the reduction was 189 PCC per 100 000 population-years (Table 3), ranging from 98 to 359 PCC per 100 000 across 13 countries. The ORs for PCC in adults was 0.89 (Table 3) and ranged from 0.60 to 0.91 (UK OR [95% CI]=0.41 [0.19; 0.86]; Figure 2).

**Table 3.** PCC per 100,000 Population-years and OR with Evaluated versus Current Strategy, Model versus UK 2014–2015 Outcomes

| Age Group | Outcome | Current Strategy | Evaluated Strategy | Odds Ratio Evaluated vs Current |
|---|---|---|---|---|
| 5–10 year-old (target) | Rate (pooled 13 countries) | 185.7 | 70.6 | 0.38 |
| | Country range: minimum | 70.1 | 23.3 | 0.18 |
| | Country range: maximum | 324.0 | 135.8 | 0.48 |
| | *UK 2014–2015* | *266.9* | *19.7* | *0.06 [0.01; 0.62]* |
| Adults ≥18 (non target) | Rate (pooled 13 countries) | 1688.3 | 1499.7 | 0.89 |
| | Country range: minimum | 615.9 | 367.6 | 0.60 |
| | Country range: maximum | 2623.6 | 2398.2 | 0.91 |
| | *UK 2014–2015* | *508.1* | *219.1* | *0.41 [0.19; 0.86]* |

Current strategy: current vaccination coverage of high-risk individuals (country-specific rates), using QIV
Evaluated strategy: Current strategy + QLAIV in 56.8% of 5–10 year-old children (all countries)
UK 2014–2015: Current strategy = non pilot areas; Evaluated strategy = 'primary school age children' area[8]
Rate per 100 000 population-years calculated using the exposed population in the mid-point of the evaluation period and range (2015–2025)
PCC: primary care consultation

**Figure 1.** OR for PCC in Target Population (children aged 5–10 years), Model versus UK 2014–2015 Outcomes

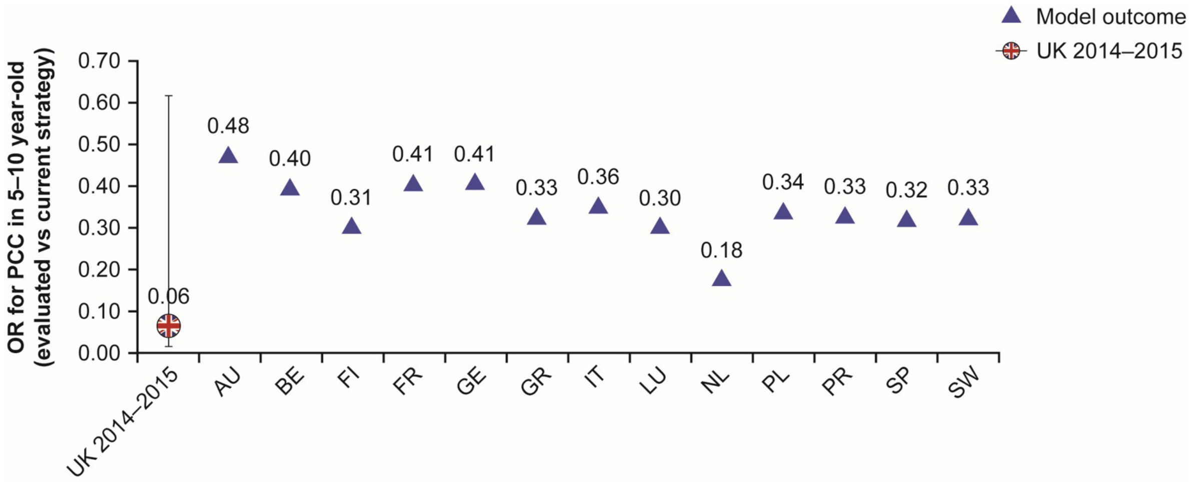

AU: Austria; BE: Belgium; FI: Finland; FR: France; GE: Germany; GR: Greece; IT: Italy; LU: Luxembourg; NL: The Netherlands; PL: Poland; PT: Portugal; SP: Spain; SW: Sweden; UK: United Kingdom

OR: odds ratio; PCC: primary care consultation

This chart shows the OR for PCC in the target population (children aged 5–10 years) modeled for 13 European countries, and compared to a similar outcome measured in the United Kingdom for season 2014–2015. The model-based OR for PCC in 5–10 year-old children ranged from 0.18 to 0.48 across 13 countries; this range falls within the 95% confidence interval of the UK results for season 2014–2015, which was [0.01–0.62].

**Figure 2.** OR for PCC in Non-target Population (≥18 year-old), Model versus UK 2014–2015 Outcomes

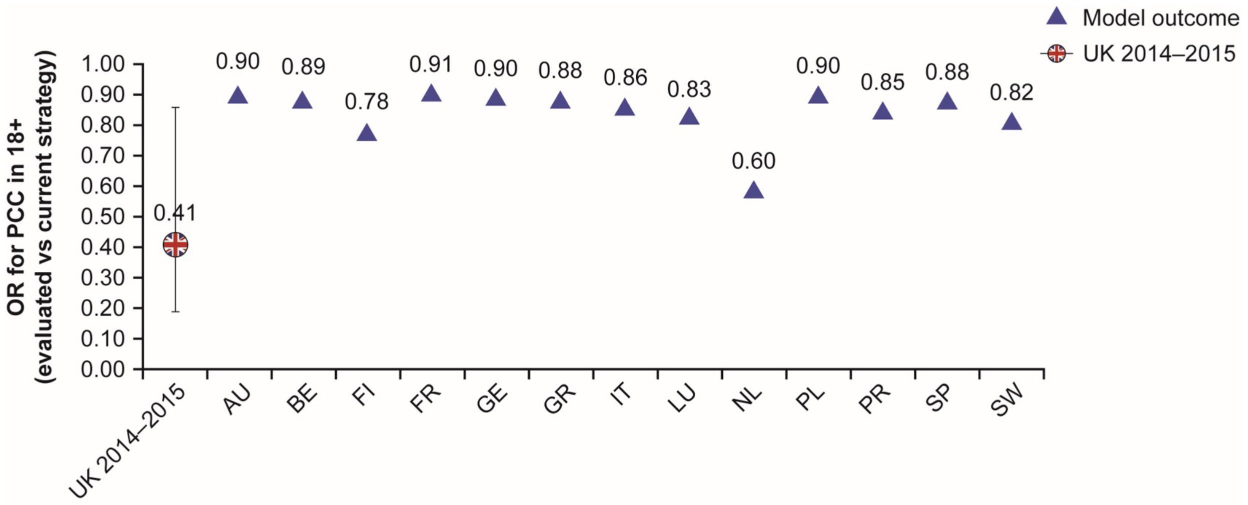

AU: Austria; BE: Belgium; FI: Finland; FR: France; GE: Germany; GR: Greece; IT: Italy; LU: Luxembourg; NL: The Netherlands; PL: Poland; PT: Portugal; SP: Spain; SW: Sweden; UK: United Kingdom

OR: odds ratio; PCC: primary care consultation

This chart shows the OR for PCC in the non-target population (adults aged 18 year-old and older) modeled for 13 European countries, and compared to a similar outcome measured in the United Kingdom for season 2014–2015. The model-based OR for PCC in adults ranged from 0.60 to 0.91 across 13 countries; this range overlaps within the 95% confidence interval of the UK results for season 2014–2015, which was [0.19–0.86].

### Number Needed to Vaccinate

Across 13 countries and 10 years, the evaluated strategy required a total of 145.9 million QLAIV doses and prevented 13.4 million PCC overall. The number of QLAIV doses needed (NNV) per averted one PCC in the total population was 11, and ranged from 6 to 19 across 13 countries. The NNV reported in the UK analysis of season 2014–2015 was 16 per averted PCC (Table 4). In the model, cumulated over 10 years and 13 countries, 165 064 influenza-related hospitalizations were prevented in the evaluated scenario. The NNV was 884 and ranged from 530 to 1524 per averted hospitalization (UK NNV=317). For ICU admissions, the NNV was 8838 and ranged from 5298 to 15 241 per averted ICU admission (UK NNV=2205).

### Sensitivity Analyses

A 50% reduction of the PCC probability in case of symptomatic influenza increased the NNV in our model to 22 and the country range to 12–37. This scenario, assuming PCC rates of 17% in low risk individuals and 33% in 1 year-old children and high risk individuals, is still encompassing the observed UK NNV value for 2014–2015 (16 doses per averted PCC).

When increasing the base case PCC probability by 50%, the model-based NNV decreased to seven (13-country range: 4–12).

For hospitalizations, the model-based NNV increased to 1768 (13-country range 1060–3048) when assuming hospitalization rates which were 50% lower than in the base case. The NNV decreased to 589 (353–1016) when assuming 50% higher hospitalization rates, which was closer to the observed UK NNV value for 2014–2015 (317 doses per averted hospitalization).

For ICU, the model-based NNV increased to 17 675 (13-country range 10 597–30 483) when assuming ICU rates which were 50% lower than in the base case, and decreased to 5892 (3532–10 161) when assuming 50% higher ICU rates. Both values were higher than the observed UK NNV value for 2014–2015 (2205 doses per averted ICU admission).

Varying the probabilities of medical resources used in case of symptomatic influenza only had a marginal impact on the ORs, because the targeted event (symptomatic influenza) remained rare (5563 cases per 100 000 population-years in the reference strategy and 4673 per 100 000 in the evaluated strategy).

**Table 4.** NNV with QLAIV per Prevented Event in the Total Population, Model versus UK 2014–2015 Outcomes

| Resource | Outcome | Current Strategy | Evaluated Strategy | Difference |
|---|---|---|---|---|
| QLAIV doses | | 0 | 14.59 million | 14.59 million |
| PCC | Number of cases | 86.34 million | 72.90 million | 13.50 million |
| | NNV (country range) | | | 11 (6–19) |
| | *UK 2014–2015* | | | *16* |
| Hospitalization | Number of cases | 1.17 million | 1.00 million | 0.17 million |
| | NNV (country range) | | | 884 (530–1524) |
| | *UK 2014–2015* | | | *317* |
| ICU | Number of cases | 116 854 | 100 347 | 16,506 |
| | NNV (country range) | | | 8838 (5298–15 241) |
| | *UK 2014–2015* | | | *2205* |

Current strategy: current vaccination coverage of high-risk individuals (country-specific rates), using QIV
Evaluated strategy: Current strategy + QLAIV in 56.8% of 5–10 year-old children (all countries)
UK 2014-15: Current strategy = non pilot areas; Evaluated strategy = 'primary school age children' area[8]
Rate per 100 000 population-years calculated using the exposed population in the mid-point of the evaluation period and range (2015–2025).

## DISCUSSION

Across a range of European countries, our model showed beneficial impact of a paediatric vaccination programme using QLAIV in primary school age children, by reducing the number of symptomatic influenza cases both in the target vaccinated children and in adult community around them, the non-target populations. As a consequence, the medical resources used to manage symptomatic influenza, including PCCs and hospitalizations, were reduced in children and adults, consistent with what was observed during a single season in the United Kingdom. These findings show that translating the UK experience to other European countries would provide similar public health benefits from a paediatric vaccination programme, on top of the current strategies mainly targeting at risk groups.

Most of our model-based results were more conservative than the observed outcomes, especially the ORs in the non-target population (adults): the ORs for PCC in the adult population were distributed around the upper value of the UK 95% CI (ie, 0.86, see Figure 2), ranging from 0.60 in The Netherlands to 0.91 in France. The comparatively conservative outcome of our simulation studies may – at least in part – be explained by the fact that we compare a 10-year evaluation period to a single influenza season: it has been reported that shortly after implementing a new vaccination strategy (as was the case in the United Kingdom), a combination of the pre-existing high level of natural immunity and the newly acquired vaccination-derived immunity lead to a transient period of over-optimistic results (termed as "honeymoon period"[54]).

Our simulation studies also show a few years of high yields after introducing QLAIV vaccination which gradually decline to a more moderate long-term level.

The two factors which had the largest impact on our results (see previous sensitivity analyses done with the same model[15-17]) were the basic reproduction number $R_0$ and the duration of naturally acquired immunity after influenza A infection. The values of $R_0$ were determined by calibration to observed incidence data from the 13 countries and were largely driven by the countries' demographic structures and the frequency of contacts between age groups. Similar results were obtained in a simulation study comparing influenza simulation results across countries[55]). At the country level, the largest and smallest model-based OR for PCC were observed in the countries with, respectively, the smallest and the largest values of all-year average $R_0$: 0.90 in The Netherlands (winter peak value: 1.29) and 1.28 in France (peak: 1.83).

We used high-level, simple, probabilities of medical resources used in case of symptomatic influenza, as our purpose was to raise awareness around the potential benefits of a UK-like paediatric programme across a range of demographic features, contact patterns, current vaccination coverage and local influenza incidence. Further heterogeneity concerning the influenza-related PCC or hospitalization rates was not specified by country in the current analysis. Across the country-specific sources that we consulted to assess influenza-related medical resources use, there were indeed important variations in study design (surveillance networks data, administrative/medical records, or prospective observational studies) and reporting (for example, by age and/or risk status or overall, by vaccination status or not, during a full season or only during peak influenza activity).

Our goal was to present an overall European picture of the QLAIV vaccination effect rather than a between-countries comparison. For this reason, no further comparisons or country-level interpretations were undertaken and additional, local medico-economic data would need to be collected to further investigate the return on investment of the evaluated program in a given country. In particular, some countries like The Netherlands or Sweden are taking into account not only the direct medical resources associated with influenza, but also the work productivity losses, to estimate the societal impact of preventing symptomatic cases. Lost work days caused by symptomatic influenza are indeed responsible for a huge economic burden every year in Europe[20] and this was not included in our study.

Important differences between the healthcare system in the United Kingdom and the systems across the 13 European countries represent a limitation of our comparison. For both medical resource and local data, a degree of pragmatism must be employed when assessing the level of granularity of data in the model.

Another limitation concerns the different PCC definitions used: the reported primary care consultations in the UK study include influenza-like illness whereas our model only counts PCC from confirmed influenza cases. This means that the baseline (events occurring in the 'reference' group with current vaccination strategy) was including more heterogeneous events in the UK study compared to our model. This difference in PCC definitions might impact the relative effect of the evaluated vaccination strategy versus baseline. For hospitalization and ICU, the UK 'real-life' study used the same 'confirmed influenza' definition as our simulation study. Model-based NNV appeared even more conservative compared with PCC, which indicates that our conclusions are robust to changes in influenza-event definitions.

In the Cochrane meta-analysis providing paediatric vaccine efficacy data for the model, the QLAIV efficacy against influenza infection in children was 80% (risk ratio 0.20 [0.13; 0.32]) which was based on six studies with 9175 participants. In recent seasons a lower QLAIV effectiveness against influenza A/H1N1pdm09 was observed in the United States,[56] while the effectiveness of the live vaccine was found similar to that of the inactivated vaccine in a Canadian study.[57] In view of these conflicting observations, extensive investigations are currently under way to gain an in-depth understanding of the live vaccine's efficacy and effectiveness. In the model, a reduced efficacy for QLAIV would lead to a lower number of averted influenza cases; however, the incremental benefit of extending the coverage of children would remain positive, as seen in previously published sensitivity analyses with the model.[16,17]

Based on the currently available evidence, our study shows that the vaccination of a large group of primary school age children with QLAIV in Europe could generate substantial benefits in the vaccinated paediatric population, and reduce the medical resources use in the adult population as well. These direct and indirect benefits of paediatric infuenza vaccination were observed in the United Kingdom during season 2014–2015, and our model-based predictions across 13 European countries compared favorably to the real-life UK data.

## FINANCIAL SUPPORT

This study was funded by an unrestricted grant from AstraZeneca.

## CONFLICT OF INTEREST

JH, RL and SDSM are or were employees of AstraZeneca at the time of the study. LG is an employee of QuintilesIMS which has received consulting fees from AstraZeneca. ME is a partner and shareholder of the contract research and consulting institute Epimos GmbH, which has received consulting fees and research support from AstraZeneca, Novartis, and GlaxoSmithKline.

## AUTHORSHIP

ME conceptualized the study, carried out the simulations and interpreted the results. LG provided local data inputs, analysed the simulation results, and drafted the manuscript. JH, RL and SDSM provided expertise and guidance on data input and assumptions. All authors critically appraised, corrected and approved the manuscript before submission.

## Figures and Tables

**Figure:** Supplementary Content

